# 29 immune-related genes pairs signature predict the prognosis of cervical cancer patients

**DOI:** 10.1038/s41598-020-70500-5

**Published:** 2020-08-25

**Authors:** Han Nie, Fanqin Bu, Jiasheng Xu, Taoshen Li, Jun Huang

**Affiliations:** 1 Department of Vascular Surgery, The Second Affiliated Hospital of Nanchang University, No. 1 Minde Road, Nanchang, 330006 Jiangxi Provence China; 2 Department of Gastrointestinal Surgery, The Second Affiliated Hospital of Nanchang University, No. 1 Minde Road, Nanchang, 330006 Jiangxi Provence China

**Keywords:** Cancer genetics, Tumour biomarkers

## Abstract

To screen the key immune genes in the development of cervical cancer, construct immune related gene pairs (IRGPs), and evaluate their influence on the prognosis of cervical cancer. Tumor Genome Atlas (TCGA) database and geo database were downloaded as training set and validation set respectively, and immune related gene data were downloaded from immport. IRGPs model is established by machine learning, and the model is analyzed and evaluated. Using the Uclcan to analyze the immune genes expression in cervical cancer, and to further explore the association with the expression level and the clinical stage and prognosis of cervical cancer. According to the analysis of training set, we identified 29 IRGPs as key gene pairs and constructed the model. The AUC value of the model was greater than 0.9, and the model group survival rate was conspicuous different (*P* < 0.001). The reliability of the model was confirmed in the validation group. Our IRGPs play an important role in the occurrence and development of cervical cancer, and can be used as a prognostic marker and potential new target of cervical cancer.

## Introduction

Cervical cancer is one of the four most common gynecological tumors^1^. Every year, at least 569,847 women in the world are diagnosed with cervical cancer, and more than 311,365 people are killed^2^. In recent years, the incidence rate of cervical cancer has decreased significantly through universal screening and health knowledge. However, the incidence rate of cervical cancer is still high in developing countries^3^. For women with low education in less developed areas, the coverage rate of cervical cancer screening is still very low^4^. Squamous cell carcinoma is the most common type of cervical cancer, accounting for 75% of cervical cancer cases, while adenocarcinoma only accounts for about 20%. In developing countries, 70% of cervical cancer patients have local infiltration or metastasis, which has led to the high mortality of cervical cancer in developing countries. Early cervical cancer is usually treated by radical hysterectomy. When there are risk factors such as lymph node metastasis and endometriosis that may lead to recurrence, they will be treated with chemotherapy^5^. The standard treatment for patients with locally advanced cervical cancer is conventional radiation therapy(CRT)^6^. The five-year survival rate of patients with locally advanced cervical cancer can be as high as 75–85% after surgical resection, radiotherapy, chemotherapy, CRT, and so on^7^. However, at present, all treatment methods are not effective for patients with paraaortic lymph node metastasis, and their three-year progression free survival time (PFS) and total survival time (OS) are 34% and 39%^8,9^, respectively. The five-year survival rate of cervical cancer patients with recurrence and metastasis was as low as 15%. Limited treatment is the main reason for this situation. Now, palliative chemotherapy is the most commonly used for patients with metastatic and recurrent cervical cancer^10^. The median survival time of patients with metastatic or recurrent cervical cancer treated with platinum/taxane chemotherapy and bevacizumab can be extended to 17 months^11^. However, these treatments are far from enough for most locally advanced and metastatic cervical cancer patients with positive lymph node metastasis.

In recent years, immunotherapy has been developed and increasingly used in cancer patients. For example, PD-L1 is overexpressed in a variety of tumor cells, including liver cancer cells and lung cancer cells, and plays an important role in regulating the immune response of tumor cells^12–15^. Currently, there are several clinical trials involving FDA-approved immunosuppressive checkpoint inhibitors, which attack tumor cells expressing PD-L1 by blocking the PD-L1/PD-1 signaling pathway, so as to improve the treatment and prognosis of patients. From the current situation, immunosuppressive therapy has achieved good results in many solid tumors^16^. The results of PD-1/PD-L1 inhibition in cervical cancer are also satisfactory. However, at present, immunoassay sites with a therapeutic effect are scarce, and the research on tumor immunotherapy is far from sufficient. In this study, we screened immune genes that are significantly related to the prognosis of cervical cancer, constructed an immune gene pair (IRGP) model based on these genes, and used it to verify the unique prognostic markers of cervical cancer.

## Method

### Data acquisition

Gene expression profile data of 255 patients with cervical squamous cell carcinoma were obtained from cancer and tumor gene map (TCGA https://www.tcga.org), and gene expression profile data set gse44001^16^ was obtained from gene expression compilation (GEO https://www.ncbi.nlm.nih.gov/geo/) database, including 300 samples of cervical cancer patients.

### Acquisition of sample immune gene expression

2,498 immune related genes were downloaded from immport (https://www.immport.org/home), including antigen presenting cells, chemokines and their receptors, cytokines and their receptors, interferon, interleukin, etc. Using limma package in R (3.61), we compared the gene expression data of cervical cancer samples downloaded from TCGA database and geo database as training set and verification set with immune related genes, and extracted the expression amount of immune related genes in cervical cancer samples.

### Construction of immune related gene pairs (IRGPs)

In the two groups of data processed in the previous step, the IRGP of the sample is calculated, and the relatively high change is selected according to the standard of media absolute deviation > 0.5. IRGP values are calculated by comparing gene expression levels in specific samples or profiles in pairs. The immune related genes are matched to compare the IRGPs. If the first IRG is larger than the second IRG, the output of the IRGP is 1; otherwise, the output is 0. If the ratio of IRGP score of 0 or 1 in training set and verification set is higher than 80%, then remove the IRGP and retain the remaining IRGP as candidate IRGP for prognosis prediction. The logistic rank test was used to screen the prognosis IRGP (FDR < 0.01). Cox risk regression analysis and glment in R (3.61) were used to perform tenfold cross validation to analyze the candidate IRGP and obtain the IRGP index. We constructed the best 29 gene pairs as immune gene pair model. We use ROC to calculate the optimal cutoff value of IRGP index, and use it as the basis to distinguish high and low risk groups.

### IRGPs model validation

The single factor and multi factor Cox proportional risk analysis and survival analysis of TCGA and gse44001 cervical cancer samples were carried out with IRGPs model.

### Infiltration of immune cells in cervical cancer samples.

In order to study the infiltration of immune cells in the high and low risk groups of cervical cancer, we used CIBERSORT^17^ to evaluate and predict the enrichment of immune cells in the samples. CIBERSORT is a tool for deconvolution of the expression matrix of immune cell subtypes based on the principle of linear support vector regression. RNA SEQ data can be used to estimate the infiltration of immune cells. CIBERSORT can analyze the relative abundance of 22 immune infiltrating cells in each sample, including NK cells, T cells, B cells and macrophages.

### Functional enrichment analysis of GSEA, go and KEGG

Gene set enrichment analysis(GSEA) enrichment analysis was carried out for each gene related to immune prognosis using the fgsea package in R (3.61)^18^ .Cluster profiler^19^ was used to enrich Gene ontology(GO)function and KEGG pathway. Significant enrichment criteria: the absolute value of NES is greater than 1, the nomp value is less than 0.05, and the fdrq value is less than 0.25.

### Expression of immune gene in cervical cancer

Ualcan ^20^ were used to analyze the expression of immune genes in cervical cancer.

### Statistical analysis

Measured data were expressed as mean ± standard deviation (x ± s) and data were compared using t test. Kaplan Meier method was used for survival analysis. The receiver operating characteristic curve (ROC curve) and ROC analysis were completed by survivalROC(1.0.3). Single factor and multi factor analysis using Cox proportional risk regression model. *P* < 0.05 was statistically significant, *P* < 0. 01 as the difference has very significant statistical significance.

### Ethical approval and consent to participate

This article does not contain any studies with patients or animals performed by any of the authors.

## Results

### Expression of immune related genes and construction of IRGPs in cervical cancer samples

We obtained gene expression data of 255 cervical cancer samples from TCGA database as training set, 300 cervical cancer samples from gse44001 as verification set, 2,498 immune related genes from immport, and 479 immune related genes from cervical cancer samples by comparing the two. Through these 479 immune related genes, we constructed 23,355 IRGPs. We remove more than 80% of the IRGP with a score of 0 or 1 from the training set and validation set, leaving 12,379 IRGP as candidates. Combine TCGA clinical data with training set data (Table 1). 73 prognosis related IRGPs were screened by lasso Cox proportional risk regression analysis. After 1,000 iterations, we selected 29 optimal IRGPs to build the immune prognosis model (Table 2).

**Table 1:** TCGA clinical data.

TCGA clincial data		
**Age**		
> 20, < 40	84	25
≥ 40, < 60	157	62
≥ 60	66	18
**Grade**		
G1	18	7
G2	136	45
G3	120	52
G4	1	1
**T**		
T1	141	70
T2	72	27
T3	21	6
T4	10	2
**M**		
M0	116	98
M1	10	7
**N**		
N0	135	78
N1	60	27

**Table 2:** Model information about IRGPI.

IRG1	Immune processes	IRG2	Immune processes	Coefficient
APOBEC3H	Antimicrobials	BTC	Cytokines	–0.305231955
ARG2	Antimicrobials	CLCF1	Cytokines	–0.260241538
BTC	Cytokines	IL16	Cytokines	0.179676189
CCL2	Cytokines	FGFR3	Cytokine_Receptors	0.145276664
CCL20	Cytokines	APOBEC3C	Antimicrobials	0.031198785
CCL20	Cytokines	ARAF	NaturalKiller_Cell_Cytotoxicity	0.057527357
CCL20	Cytokines	PLXNA1	Chemokine_Receptors	0.140136903
CCL28	Cytokines	MAP3K14	TCRsignalingPathway	0.073050543
CXCL1	Cytokines	TNFSF10	TNF_Family_Members	0.091865234
CXCL2	Cytokines	PTAFR	Chemokine_Receptors	0.055416654
DES	Cytokines	EPOR	Cytokine_Receptors	–0.176178789
DES	Cytokines	VEGFC	Cytokines	–0.109471751
DLL4	Antimicrobials	DES	Cytokines	0.157519455
FLT3LG	Cytokines	INHBA	TGFb_Family_Member	–0.410075729
HCK	Antimicrobials	SAA2	Chemokines	–0.131734804
HCK	Antimicrobials	STC2	Cytokines	–0.150239991
HLA-DQA2	Antigen_Processing_and_Presentation	LTB4R2	Cytokine_Receptors	–0.145752601
IL1B	Antimicrobials	DUOX1	Antimicrobials	0.222307011
IL1B	Antimicrobials	EDN1	Chemokines	0.357205394
JAK1	Antimicrobials	APOBEC3C	Antimicrobials	0.379893592
NOD1	Antimicrobials	CSF2RB	Cytokine_Receptors	0.123869391
NRP1	Cytokine_Receptors	CD3D	TCRsignalingPathway	0.172301934
PLXNB3	Cytokine_Receptors	FGFR2	Cytokine_Receptors	0.396666512
PSMD7	Antigen_Processing_and_Presentation	SHC1	NaturalKiller_Cell_Cytotoxicity	–0.246365863
RBP7	Antimicrobials	CXCR3	Chemokine_Receptors	0.198196775
RBP7	Antimicrobials	DES	Cytokines	0.495839154
STC1	Cytokines	TNFRSF18	Cytokine_Receptors	0.093742914
TLR3	Antimicrobials	CXCR6	Antimicrobials	0.315887096
VAV3	BCRSignalingPathway	NRP1	Cytokine_Receptors	–0.36820747

### IRGPs model validation

**Figure 1:**
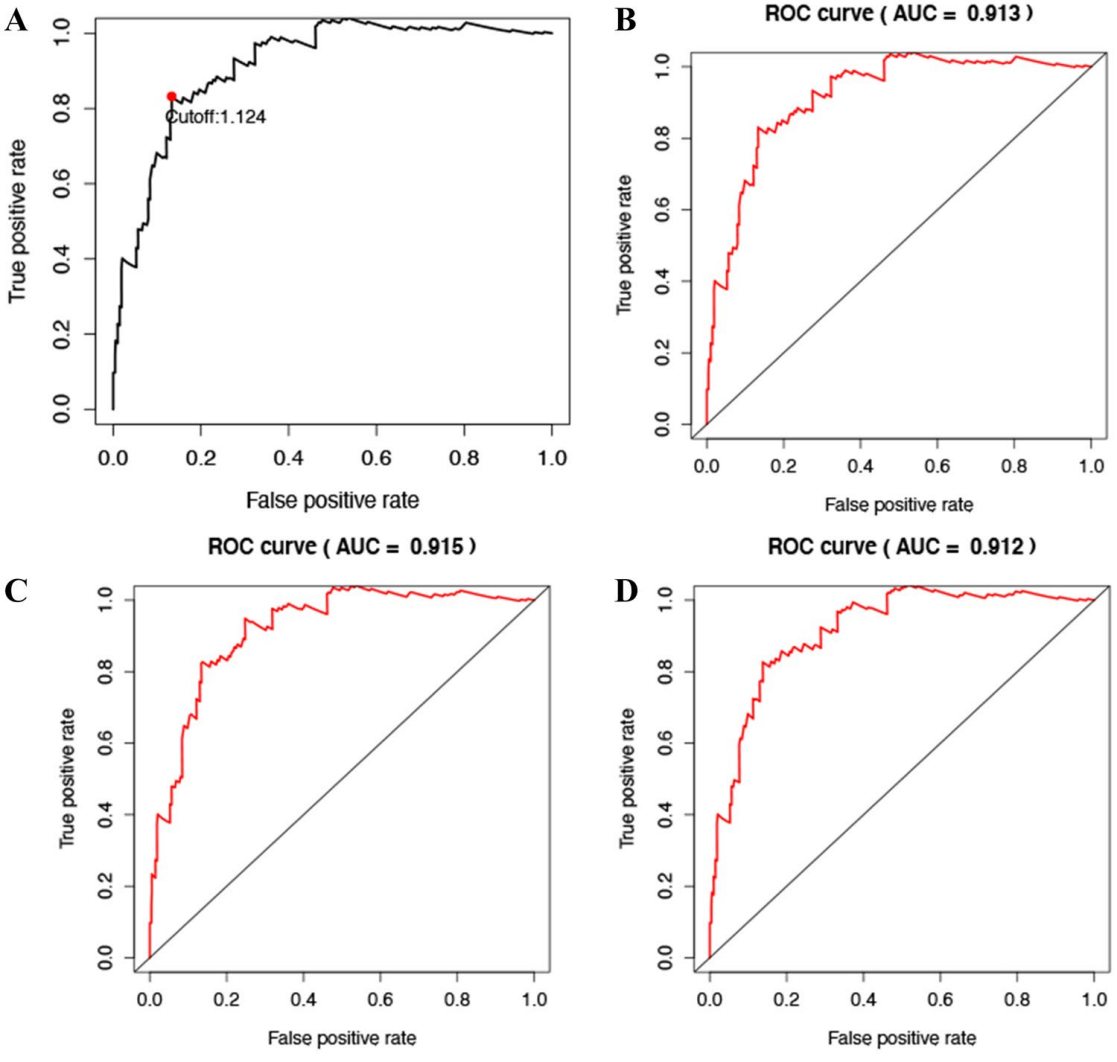
(**A**) Time-dependent ROC curve for IRGPI in the training cohort. (**B)** Time-dependent ROC curve for IRGPI in 1 year. (**C)** Time-dependent ROC curve for IRGPI in 3 year. (**D**) Time-dependent ROC curve for IRGPI in 5 year.

**Figure 2:**
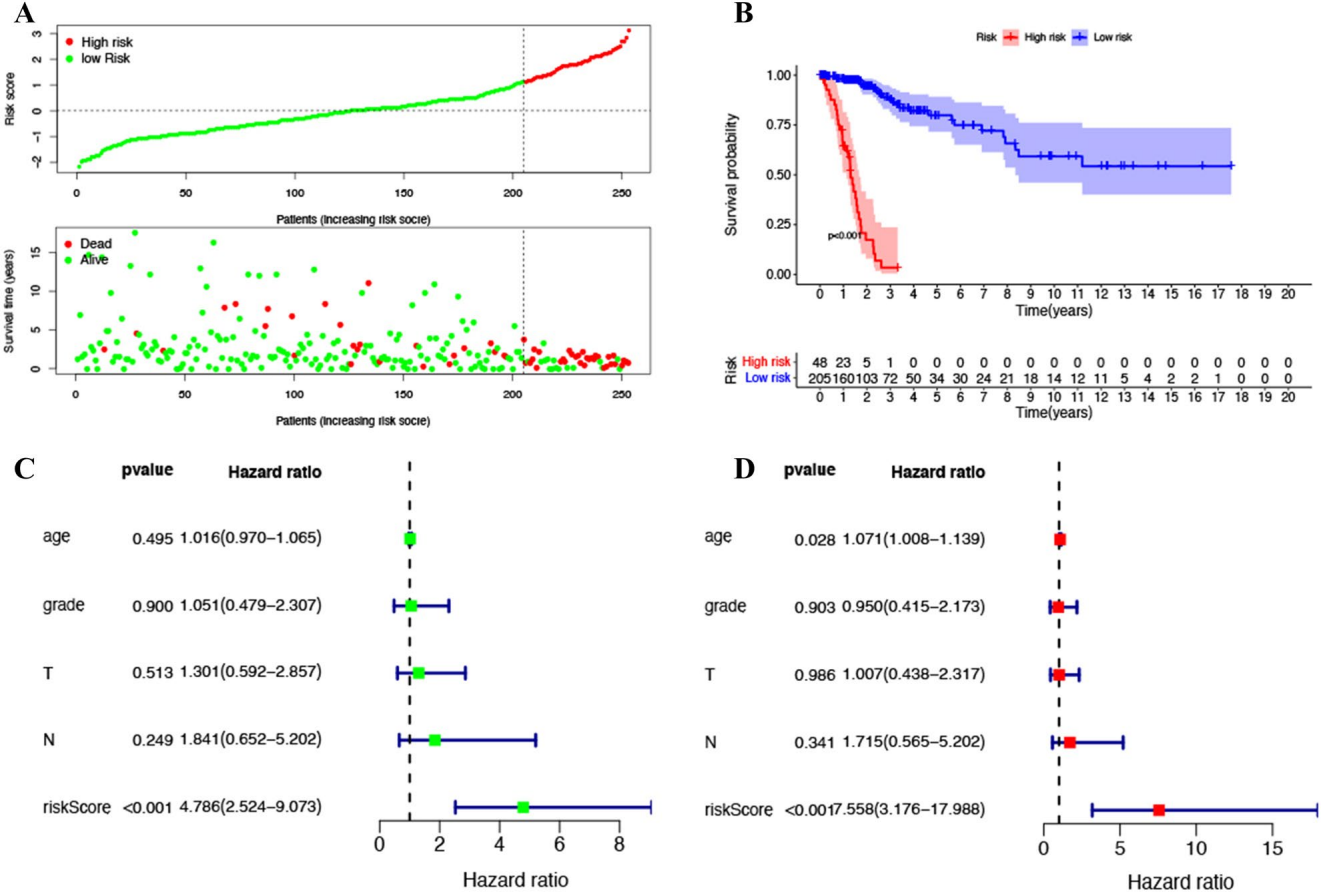
(**A**) The model divides the training set patients into low-risk or high-risk groups. (**B)** Kaplan Meier curve between high and low risk groups. (**C)** Training set single factor Cox regression analysis forest map. (**D)** Training set multivariate Cox regression analysis forest map.

**Figure 3:**
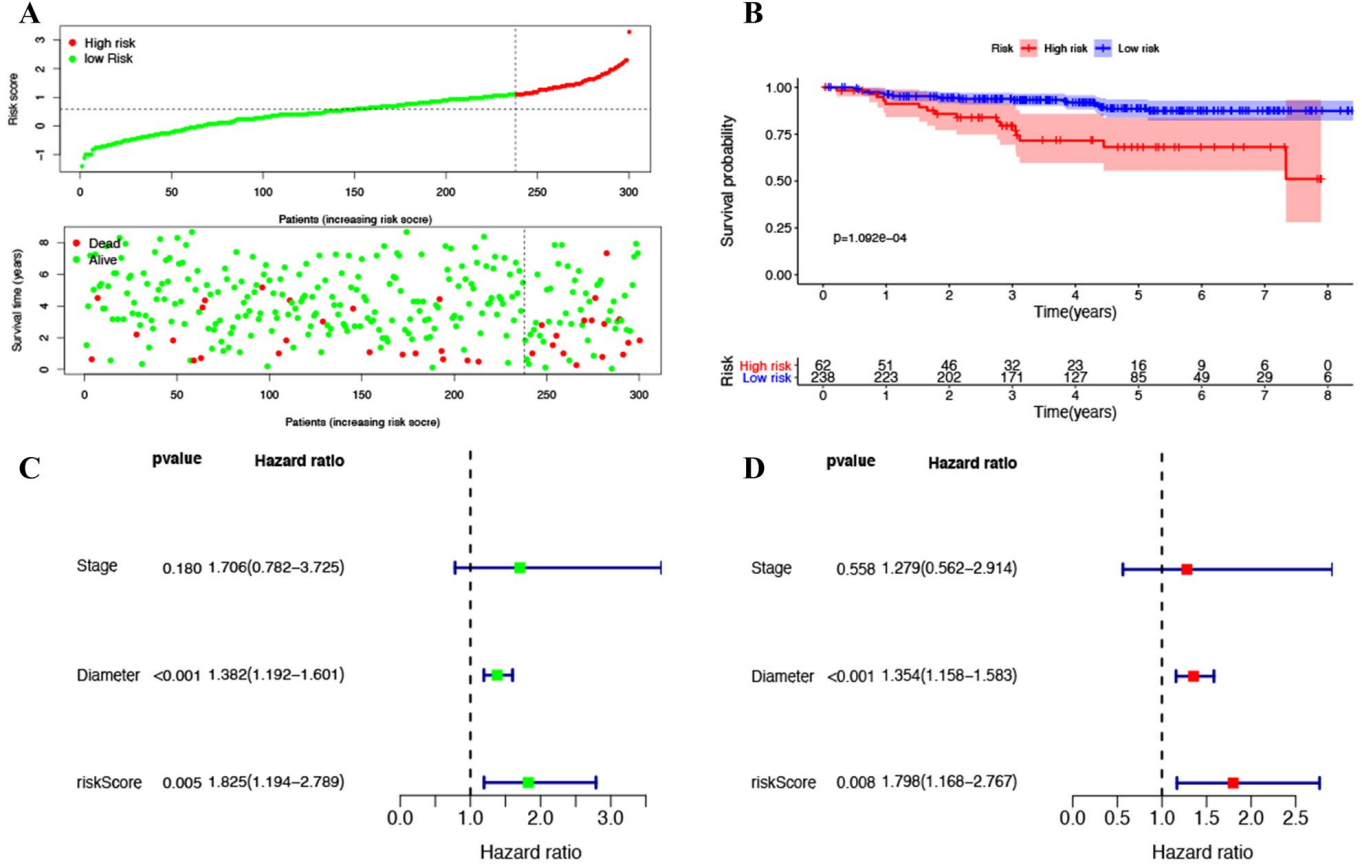
(**A)** The model divides the validation set patients into low-risk or high-risk groups. (**B)** Kaplan Meier curve between high and low risk groups. (**C)** Validation set single factor Cox regression analysis forest map. (**D**) Validation set multivariate Cox regression analysis forest map.

The immune prognosis model was applied to the training set, and the patients in each training set were scored. According to ROC curve analysis, the optimal cutoff value for patients to be divided into high and low risk groups is 1.124 (Fig. 1A). After evaluating the model, we found that AUC value of model 1, 3 and 5 years is 0.913 (Fig. 1B), 0.913 (Fig. 1C) and 0.912 (Fig. 1D). The results show that our immune prognosis gene has a high reliability for the model. The training set was divided into high-risk group (Fig. 2A) and high-risk group (Fig. 2B). The results showed that the overall survival rate (OS) of high-risk group was significantly lower than that of low-risk group. For TCGA training set data, single factor and multi factor Cox risk regression analysis showed that only IRGPs model showed significant prognostic effect in single factor Cox (Fig. 2C), while age and IRGPs could be significant independent prognostic factors in multi factor Cox (Fig. 2D). Applying this model to the validation set of gse44001 (Fig. 3A) (Table 3), survival analysis showed that the OS of patients in the high-risk group was significantly lower than that in the low-risk group (Fig. 3B). In the single factor and multi factor Cox analysis, IRGPs model and tumor size were significantly correlated with prognosis (Fig. 3C,D).

**Table 3:** GSE44001 clincial data.

GSE44001 clincial data	
Stage	
1	258
2	42
Largest diameter (cm)	
< 2	81
≥ 2, < 4	137
≥ 4, < 6	65
≥ 6	17

### Infiltration of immune cells in cervical cancer samples

**Figure 4:**
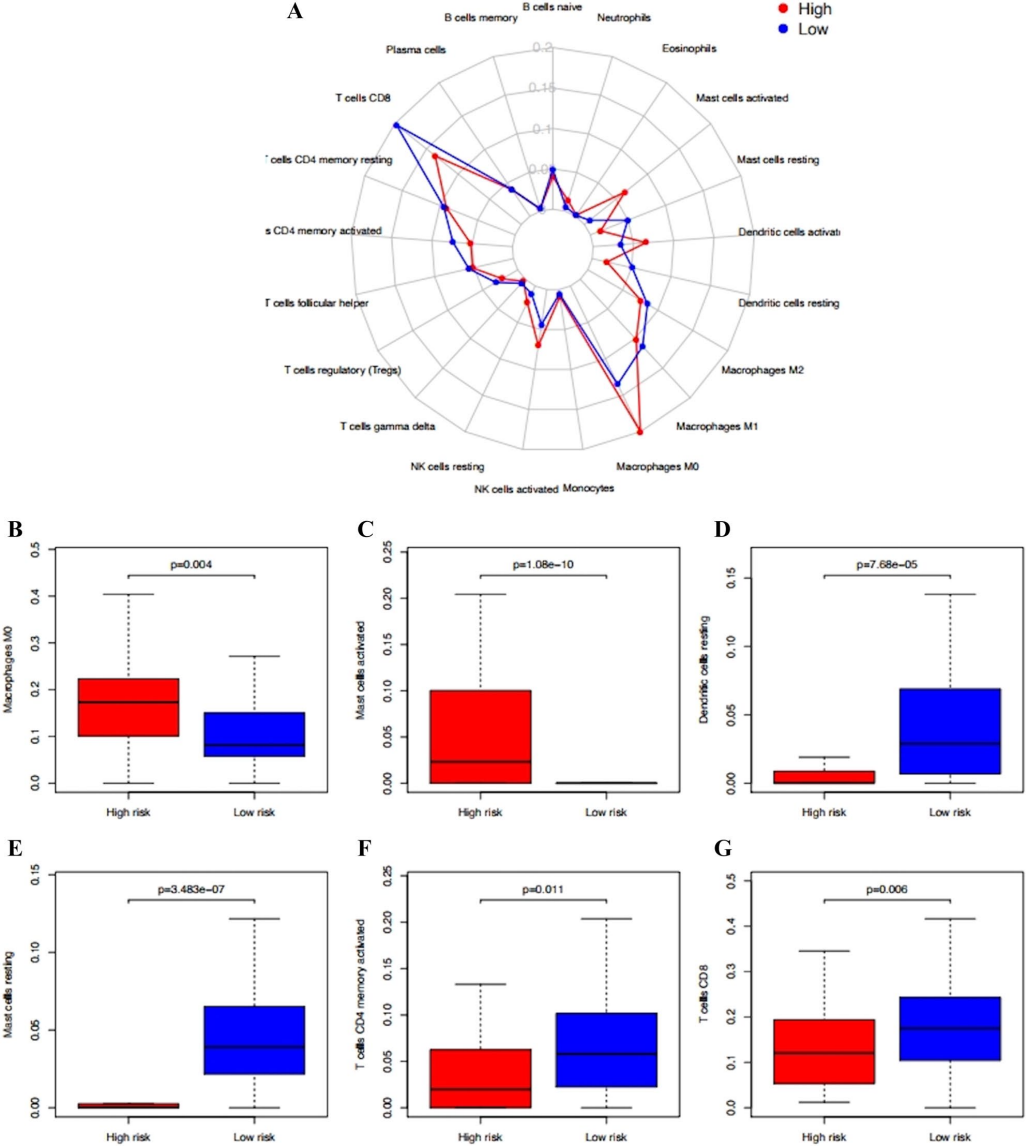
(**A**) Immune infiltration status within IRGPI risk groups. (**B**) Expression of Macrophage M0. (**C**) Expression of Mast cells activated. (**D**) Expression of Dendritic cells resting. (**E**) Expression of Mast cells resting. (**F**) Expression of T cells CD4 memory activated. (**G**) T cells CD8.

Most studies believe that the occurrence and development of tumor are closely related to immune cells, so it is an ideal method to study the infiltration of immune cells in tumor. We used CIBERSORT to analyze the infiltration of 22 kinds of immune cells in patients with high and low risk groups. Figure 4A shows the expression of immune cells in different risk groups. Macrophage M0 (Fig. 4B), activated mast cells (Fig. 4C) were significantly overexpressed in the high-risk group, while stationary dendritic cells (Fig. 4D), stationary mast cells (Fig. 4E), activated CD4T cells (Fig. 4F), and cd8t cells (Fig. 4G) were overexpressed in the low-risk group.

### Functional enrichment analysis of GSEA, go and KEGG

**Figure 5:**
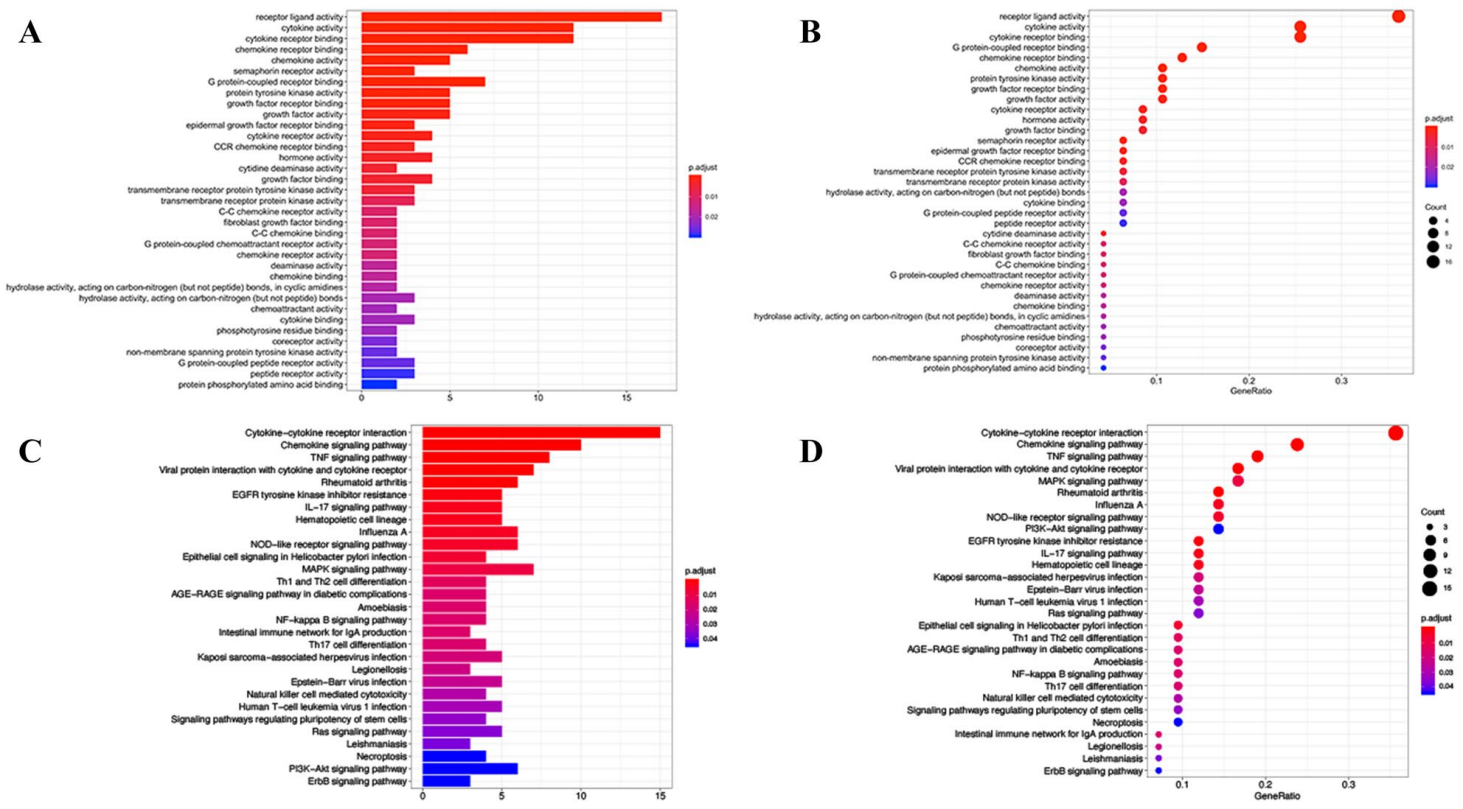
(**A)** Histogram graph of Immune-related genes GO analysis results. (**B**) Point graph of Immune-related genes GO analysis results. (**C**) Histogram graph of Immune-related genes KEGG pathway analysis results. (**D**) Point graph of Immune-related genes KEGG pathway analysis results.

**Figure 6:**
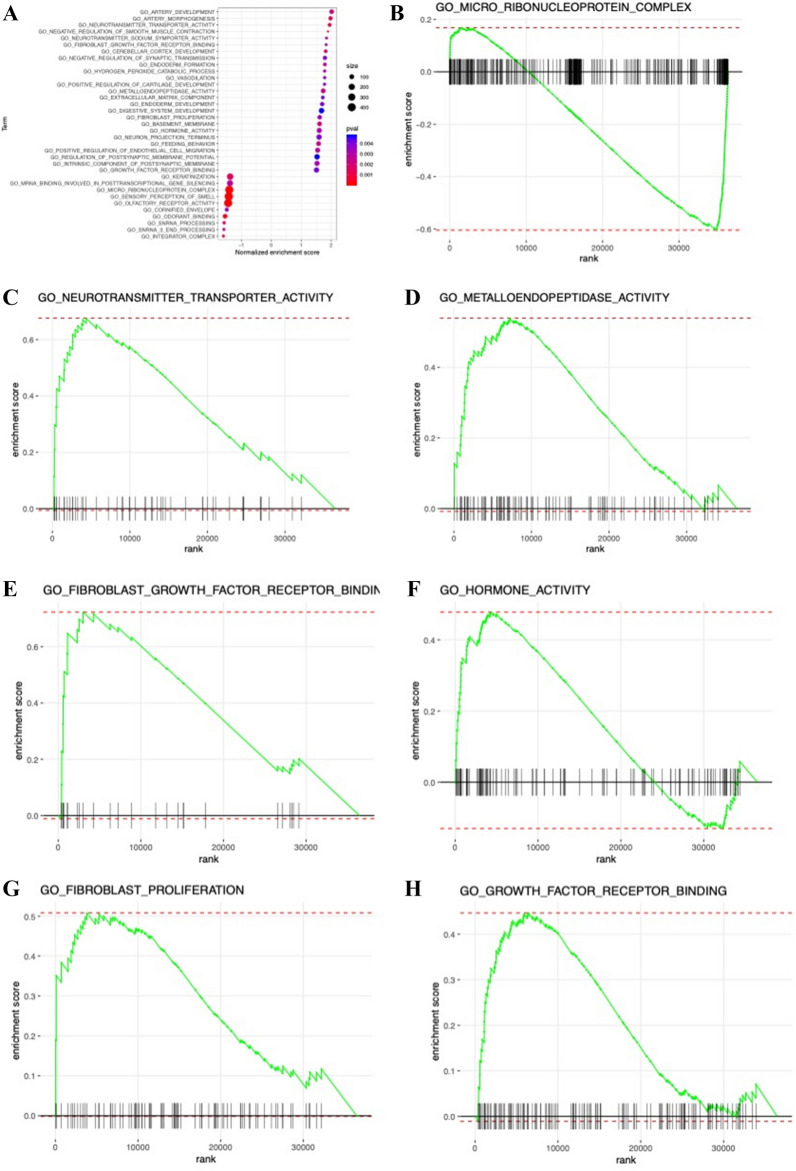
(**A**) GSEA analysis of 47 immune signature genes. (**B**–**H**) In the high immune risk group of cervical cancer, 7 cancer marker genes were abundant (*P* < 0.05, FDR < 0.25).

We analyzed the function enrichment of IRGP in the model. The results of go analysis showed that IRGP in the model was mainly enriched in the binding of cytokines and their receptors, the binding of chemokines and their receptors, the binding of growth factors and their receptors, the binding of epidermal growth factor receptors, the binding of fibroblast growth factors and the activity of tyrosine kinase (Fig. 5A,B). KEGG results showed that these IRGP were involved in cytokine cytokine receptor interaction, chemokine signaling, tumor necrosis factor signaling, MAPK signaling, NF kappa B signaling, natural killer cell-mediated cytotoxicity, viral proteins and cytokines, and Th1 and Th2 cell differentiation (Fig. 5C,D). The results of GSEA (Fig. 6A) showed that these IRGP were significantly enriched in trace ribonucleoprotein complex (Fig. 6B), neurotransmitter transporter activity (Fig. 6C), endopeptidase activity (Fig. 6D), fibroblast growth factor receptor binding (Fig. 6E), hormone activity (Fig. 6F), fibroblast cell proliferation (Fig. 6G), and growth factor receptor binding (Fig. 6H).

### Expression of immune gene in cervical cancer

**Figure 7:**
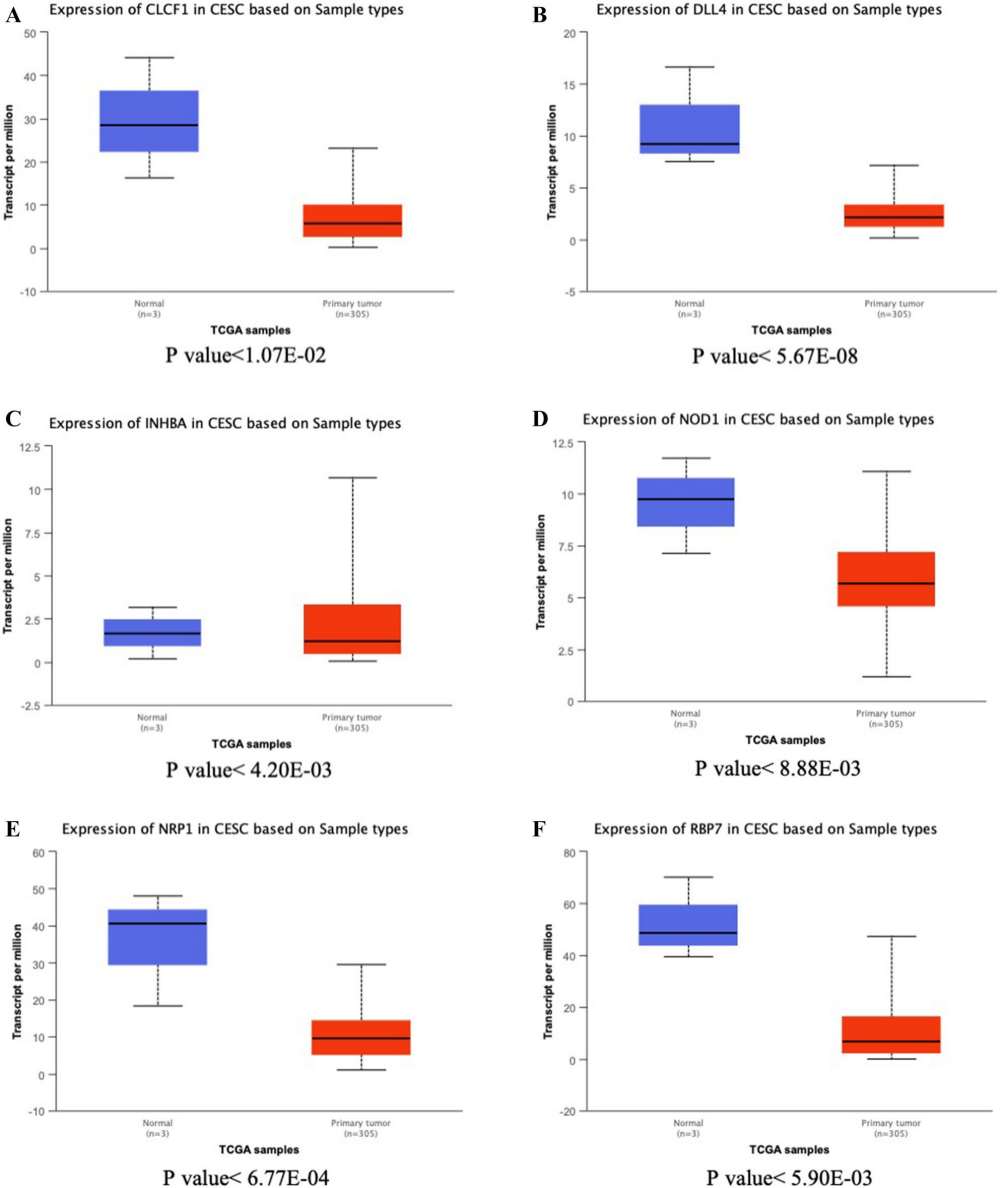
(**A**) Expression of CLCF1 in cervical cancer and normal tissues. (**B**) Expression of DLL4 in cervical cancer and normal tissues. (**C**) Expression of INHBA in cervical cancer and normal tissues (**D)** Expression of NOD1 in cervical cancer and normal tissues. (**E**) Expression of NRP1 in cervical cancer and normal tissues. (**F)** Expression of RBP7 in cervical cancer and normal tissues.

**Figure 8:**
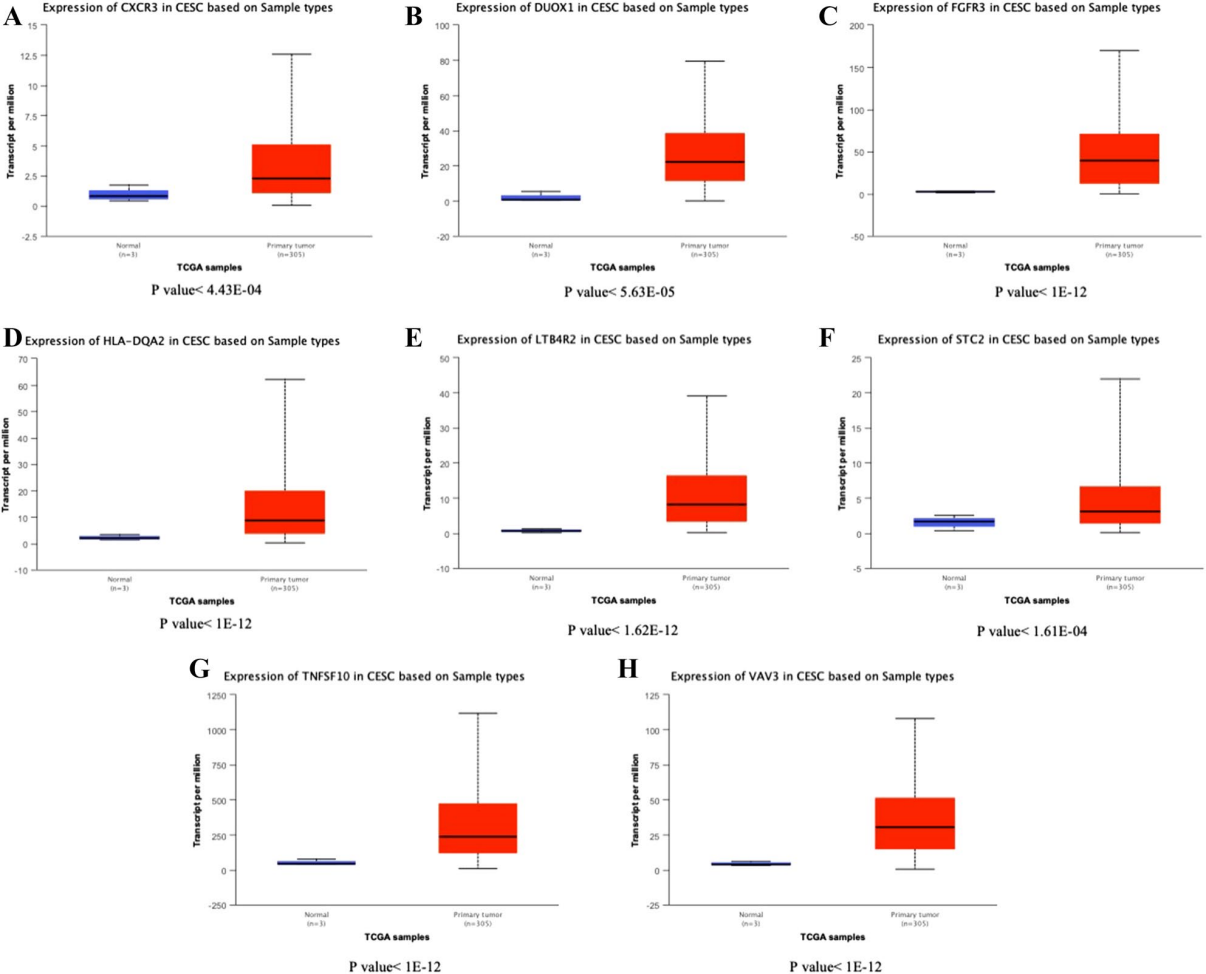
(**A**) Expression of CXCR3 in cervical cancer and normal tissues. (**B**) Expression of DUOX1 in cervical cancer and normal tissues. (**C**) Expression of FGFR3 in cervical cancer and normal tissues. (**D**) Expression of HLA-DQA2 in cervical cancer and normal tissues. (**E**) Expression of LTB4R2 in cervical cancer and normal tissues. (**F**) Expression of STC2 in cervical cancer and normal tissues. (**G**) Expression of TNFSF10 in cervical cancer and normal tissues. (**H**) Expression of CESC in cervical cancer and normal tissues.

**Figure 9:**
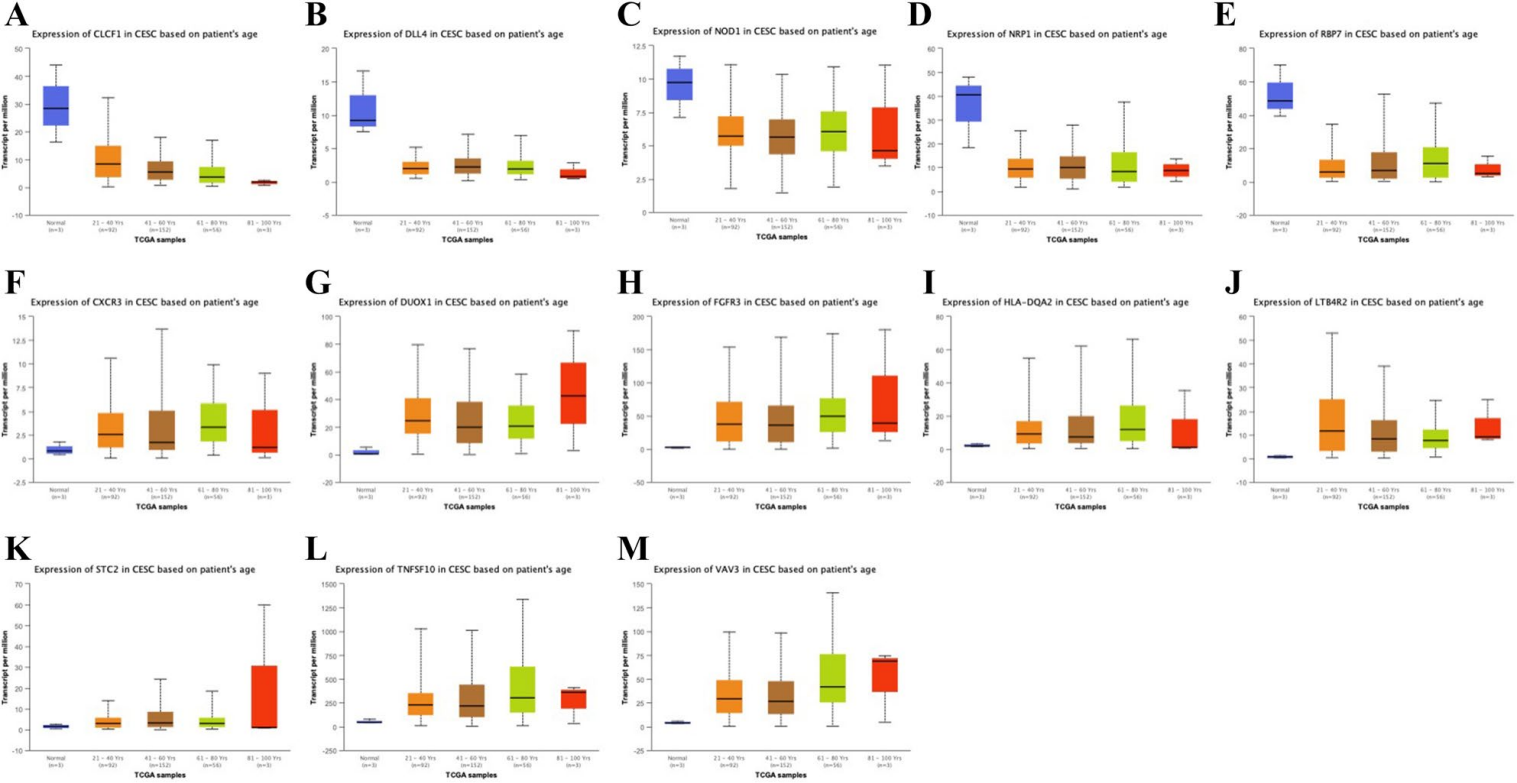
(**A**) Expression of CLCF1 in cervical cancer and normal tissues. at different ages. (B) Expression of DLL4 in cervical cancer and normal tissues at different ages. (**C)** Expression of NOD1 in cervical cancer and normal tissues at different ages. (**D**) Expression of NRP1 in cervical cancer and normal tissues at different ages. (**E**) Expression of RBP7 in cervical cancer and normal tissues at different ages. (**F**) Expression of CXCR3 in cervical cancer and normal tissues at different ages. (**G**) Expression of DUOX1 in cervical cancer and normal tissues at different ages. (**H**) Expression of FGFR3 in cervical cancer and normal tissues at different ages. (**I**) Expression of HLA-DQA2 in cervical cancer and normal tissues at different ages. (**J**) Expression of LTB4R2 in cervical cancer and normal tissues at different ages. (**K**) Expression of STC2 in cervical cancer and normal tissues at different ages. (**L**) Expression of TNFSF10 in cervical cancer and normal tissues at different ages. (**M**) Expression of VAV3 in cervical cancer and normal tissues at different ages.

**Figure 10:**
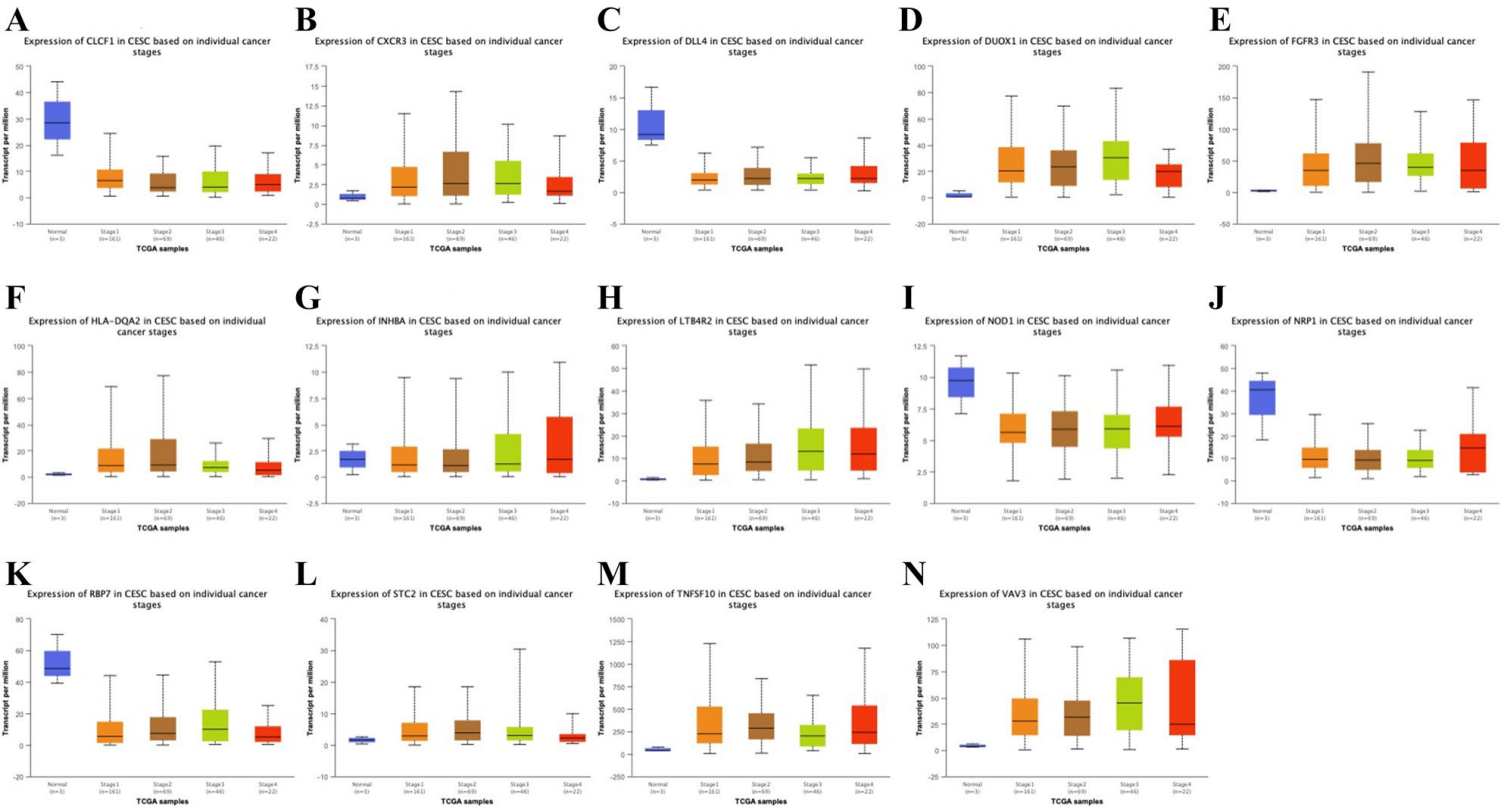
(**A**) Expression of CLCF1 in cervical cancer and normal tissues. at different stages. (**B**) Expression of CXCR3 in cervical cancer and normal tissues at different stages. (**C**) Expression of DLL4 in cervical cancer and normal tissues at different stages. (**D**) Expression of DUOX1 in cervical cancer and normal tissues at different stages. (**E**) Expression of FGFR3 in cervical cancer and normal tissues at different stages. (**F**) Expression of HLA-DQA2 in cervical cancer and normal tissues at different stages. (**G**) Expression of INHBA in cervical cancer and normal tissues at different stages. (**H**) Expression of LTB4R2 in cervical cancer and normal tissues at different stages. (**I**) Expression of NOD1 in cervical cancer and normal tissues at different stages. (**J**) Expression of NRP1 in cervical cancer and normal tissues at different stages. (**K**) Expression of RBP7 in cervical cancer and normal tissues at different stages. (**L**) Expression of STC2 in cervical cancer and normal tissues at different stages. (**M**) Expression of TNFSF10 in cervical cancer and normal tissues at different stages. (**N**) Expression of VAV3 in cervical cancer and normal tissues at different stages.

We explored the expression of IRGP in cervical cancer using the ualcan model (Table 4). There were 6 low-level expression of IRGP in cervical cancer (Fig. 7) and 8 high-level expression of IRGP (Fig. 8). There were differences in the expression of 5 low expression IRGP and 8 high expression IRGP in different age groups (Fig. 9), and there were differences in the expression of 14 IRGP in different stages of cervical cancer (Fig. 10).

**Table 4:** P value of IRGPs expression in different ages and stages.

	Age	Pval	Stage	Pval
CLCF1	Normal-vs-Age(41-60Yrs)	7.77E-05	Normal-vs-Stage2	1.85E-06
	Age(21-40Yrs)-vs-Age(41-60Yrs)	1.23E-02	Normal-vs-Stage3	2.20E-04
	Age(21-40Yrs)-vs-Age(61-80Yrs)	4.83E-04	Stage1-vs-Stage2	5.42E-03
	Age(21-40Yrs)-vs-Age(81-100Yrs)	1.15E-07	Stage1-vs-Stage3	3.04E-02
	Age(41-60Yrs)-vs-Age(61-80Yrs)	2.09E-02	Stage1-vs-Stage4	4.60E-04
	Age(41-60Yrs)-vs-Age(81-100Yrs)	4.33E-07		
DLL4	Normal-vs-Age(41-60Yrs)	2.70E-06	Normal-vs-Stage1	6.96E-07
	Normal-vs-Age(61-80Yrs)	3.54E-05	Normal-vs-Stage4	5.46E-03
	Normal-vs-Age(81-100Yrs)	2.81E-02		
INHBA	Normal-vs-Age(21-40Yrs)	9.82E-03	Normal-vs-Stage1	2.52E-03
	Normal-vs-Age(41-60Yrs)	2.60E-03	Normal-vs-Stage2	2.10E-02
	Age(21-40Yrs)-vs-Age(81-100Yrs)	3.84E-04	Normal-vs-Stage4	4.39E-02
	Age(41-60Yrs)-vs-Age(61-80Yrs)	3.22E-02		
	Age(41-60Yrs)-vs-Age(81-100Yrs)	1.63E-05		
	Age(61-80Yrs)-vs-Age(81-100Yrs)	6.00E-04		
NOD1	Normal-vs-Age(21-40Yrs)	8.68E-03	Normal-vs-Stage1	3.90E-03
	Normal-vs-Age(41-60Yrs)	1.06E-02	Normal-vs-Stage2	1.86E-02
	Normal-vs-Age(61-80Yrs)	1.09E-02	Normal-vs-Stage3	3.95E-02
			Normal-vs-Stage4	3.17E-02
NRP1	Normal-vs-Age(21-40Yrs)	1.22E-04	Normal-vs-Stage1	1.22E-03
	Normal-vs-Age(41-60Yrs)	3.96E-03	Normal-vs-Stage2	1.64E-03
	Normal-vs-Age(61-80Yrs)	2.81E-04	Normal-vs-Stage3	3.96E-04
	Normal-vs-Age(81-100Yrs)	4.59E-02	Normal-vs-Stage4	9.84E-03
RBP7	Normal-vs-Age(21-40Yrs)	4.21E-04	Normal-vs-Stage1	2.22E-02
	Normal-vs-Age(41-60Yrs)	8.76E-03	Normal-vs-Stage2	3.15E-06
	Normal-vs-Age(61-80Yrs)	4.41E-02	Normal-vs-Stage3	2.26E-02
	Normal-vs-Age(81-100Yrs)	1.05E-02	Normal-vs-Stage4	3.87E-07
			Stage1-vs-Stage4	7.34E-03
			Stage3-vs-Stage	4.33E-01
CXCR3	Normal-vs-Age(21-40Yrs)	3.84E-04	Normal-vs-Stage1	1.18E-04
	Normal-vs-Age(41-60Yrs)	5.66E-05	Normal-vs-Stage2	1.73E-05
	Normal-vs-Age(61-80Yrs)	2.18E-05	Normal-vs-Stage3	3.55E-04
			Normal-vs-Stage4	2.22E-02
DUOX1	Normal-vs-Age(21-40Yrs)	8.03E-09	Normal-vs-Stage1	1.60E-05
	Normal-vs-Age(41-60Yrs)	1.39E-06	Normal-vs-Stage2	2.59E-09
	Normal-vs-Age(61-80Yrs)	2.25E-08	Normal-vs-Stage3	1.31E-09
			Normal-vs-Stage4	2.58E-04
FGFR3	Normal-vs-Age(21-40Yrs)	1.11E-16	Normal-vs-Stage1	1.62E-12
	Normal-vs-Age(41-60Yrs)	2.04E-13	Normal-vs-Stage2	1.99E-12
	Normal-vs-Age(61-80Yrs)	7.25E-10	Normal-vs-Stage3	1.76E-05
	Age(21-40Yrs)-vs-Age(61-80Yrs)	3.05E-02	Normal-vs-Stage4	2.02E-04
HLA-DQA2	Normal-vs-Age(21-40Yrs)	4.22E-07	Normal-vs-Stage1	6.63E-12
	Normal-vs-Age(41-60Yrs)	6.93E-10	Normal-vs-Stage2	3.90E-05
	Normal-vs-Age(61-80Yrs)	1.74E-05	Normal-vs-Stage3	2.41E-04
LTB4R2	Normal-vs-Age(21-40Yrs)	4.33E-15	Normal-vs-Stage1	1.62E-12
	Normal-vs-Age(41-60Yrs)	1.62E-12	Normal-vs-Stage2	8.60E-10
	Normal-vs-Age(61-80Yrs)	5.50E-10	Normal-vs-Stage3	4.79E-09
	Age(21-40Yrs)-vs-Age(61-80Yrs)	3.41E-02	Normal-vs-Stage4	1.75E-04
			Stage1-vs-Stage3	1.48E-02
STC2	Normal-vs-Age(21-40Yrs)	8.03E-04	Normal-vs-Stage1	2.40E-04
	Normal-vs-Age(41-60Yrs)	1.59E-06	Normal-vs-Stage2	4.35E-05
	Normal-vs-Age(61-80Yrs)	4.58E-04	Normal-vs-Stage3	1.19E-04
	Age(21-40Yrs)-vs-Age(41-60Yrs)	1.92E-02	Normal-vs-Stage4	4.63E-02
TNFSF10	Normal-vs-Age(21-40Yrs)	5.24E-10	Normal-vs-Stage1	2.30E-13
	Normal-vs-Age(41-60Yrs)	4.15E-11	Normal-vs-Stage2	2.97E-09
	Normal-vs-Age(61-80Yrs)	5.59E-10	Normal-vs-Stage3	2.63E-07
			Normal-vs-Stage4	8.40E-04
			Stage1-vs-Stage3	1.70E-02
VAV3	Normal-vs-Age(21-40Yrs)	1.62E-12	Normal-vs-Stage1	1.62E-12
	Normal-vs-Age(41-60Yrs)	1.62E-12	Normal-vs-Stage2	4.93E-12
	Normal-vs-Age(61-80Yrs)	9.34E-14	Normal-vs-Stage3	2.84E-12
	Age(21-40Yrs)-vs-Age(61-80Yrs)	1.08E-02	Normal-vs-Stage4	1.25E-04
	Age(41-60Yrs)-vs-Age(61-80Yrs)	2.08E-03

## Discussion

Cervical cancer is one of the most common gynecological malignancies. HPV infection is considered to be the main cause of cervical cancer^21,22^ although the incidence rate of cervical cancer has been significantly decreased due to the development and promotion of HPV vaccine^23^. But incidence rate of cervical cancer is still high in developing countries and China's low income countries^24^. At present, for cervical cancer patients without invasion and lymphatic metastasis, the effect of surgery combined with radiotherapy and chemotherapy is better. If metastasis and infiltration occur, the treatment effect of cervical cancer patients will become very unsatisfactory. In recent years, immunotherapy has performed well in a variety of cancers including cervical cancer^25–27^. Blocking PD-L1 / PD-1 signaling pathway to attack tumor cells expressing PD-L1 is the current mainstream method^28^. Although the anticancer activity of PD-1 and PD-L1 inhibitors is exciting, such immunotherapy is not effective for all patients, and a meta-analysis shows that patients who receive PD-1 / PD-L1 inhibitors have a higher risk of rash, thyroid dysfunction, pruritus, pneumonia and colitis^29–31^. Therefore, it is of great significance for the detection and treatment of cervical cancer to predict and find more biomarkers that may be related to immune prognosis.

At present, most of the prognostic genes need to be standardized to reduce the errors caused by sequencing platform and samples. In this study, the scores of IRGPs constructed by us are calculated from the gene expression data of the same sample, which can not only ignore the impact of different platforms, but also do not need to standardize and scale the data. This method has been used in many studies, including cancer molecular classification, with high reliability^32,33^.

In this study, we screened 29 pairs of IRGP to construct the immune prognosis model related to the overall survival rate of cervical cancer patients. The AUC values of the model in 1, 3 and 5 years were all greater than 0.9. According to these 29 pairs of IRGP, they were divided into high-risk group and low-risk group. In TCGA training group and GSE44001 verification group, the OS of high-risk group was significantly lower than that of low-risk group (*P* < 0.01). These 29 pairs of IRGP have a good effect on sample discrimination. We found that macrophage Mo and activated mast cells were significantly over expressed in high-risk group by immunocyte infiltration analysis of samples. The existing research shows that mast cells and macrophages play an important role in cervical cancer, which can promote the development of cervical cancer by promoting lymphangiogenesis and angiogenesis^34–36^. However, in the low-risk group, the expression of static dendritic cells, static mast cells, activated CD4T cells and cd8t cells is high. Although the effect of CD4T cells on cervical cancer has not been agreed, the cd8t cells are closely related to the better prognosis of cervical cancer patients^37–39^, there is evidence that dendritic cells will decrease in patients with high HPV infection, which indicates that high expression of dendritic cells is beneficial to resist cervical cancer^40^, which is consistent with our results. The enrichment analysis of go and GSEA showed that these immune genes were mainly involved in the binding of cytokines and their receptors, the binding of chemokines and their receptors, the binding of growth factors and their receptors, the binding of epidermal growth factor receptors, the activity of metalloendopeptidase, the binding of fibroblast growth factors and their receptors, hormone activity, fibroblast proliferation, and the binding process of growth factor receptors.As we all know, cytokines and chemokines are the key factors in the immune response. for example: In cervical cancer, IL-10 can interfere with the differentiation of dendritic cells and thus play a strong immunosuppressive effect,TGF—β 1 can inhibit T cell proliferation and attenuate immune response^41^. Research shows that growth factors and epidermal growth factors are closely related to the growth of cervical cancer and the survival rate of cervical cancer patients. High expression of growth factors and epidermal growth factors often predict poor prognosis^42–44^. Growth of fibroblasts can stimulate angiogenesis at the early stage of tumor The proliferation and invasion of cancer cells and the remodeling of extracellular matrix promote the growth of cervical cancer^45,46^. KEGG results showed that these immune genes were mainly enriched in chemokine signaling pathway, tumor necrosis factor signaling pathway, MAPK signaling pathway, NF kappa B signaling pathway, natural killer cell-mediated cytotoxicity, viral protein and cytokine, and Th1 and Th2 cell differentiation. Th1 and Th2 may be involved in the pathogenesis and growth of cervical cancer. Th1 may be the target of predicting chemotherapy response of advanced cervical cancer^47–50^, while other pathways are classical signal pathways related to cancer. Immune cytokines play an important role in cervical lesions. Torres et al. Found that IL-10 is highly expressed in the cervix of women with persistent HPV, which may be related to the persistence of HPV and the promotion of disease progression. Further research by their team showed that copy individuals of IL-4, IL-6, IL-10 and TGFB1 were significantly associated with cervical cancer, and could be used as biomarkers for susceptibility to the disease^51,52^.These 29 pairs of IRGP have 47 different immune genes, most of which are cytokines, antimicrobial agents and natural killer cells, which are involved in various stimulation reactions and play a key role. In cervical cancer, HPV can inhibit the apoptosis of cervical cancer cells by down regulating NOD1^53^. In our sample, we also found that the expression of NOD1 in tumor tissue is low and there are differences in different ages and stages (Figs. 7D, 9C, 10I). Sang Yeon Cho et al. Found that duox1 is highly expressed in cervical squamous cell carcinoma and can play a good prognostic role by increasing the amount of innate immune cells^54^. The analysis also showed that DUOX1 is highly expressed in tumor tissues and related to age and grade (Figs. 8B, 9G, 10D). Stc2 can promote the proliferation of cervical cancer cells and increase the resistance to cisplatin^55^, while high expression of DDL4 is usually associated with low pelvic lymph node metastasis and survival rate of cervical cancer^56^. Therefore, we believe that the IRGP constructed in this study plays an important role in the development and prognosis of cervical cancer.

There are also some deficiencies in our research. Although we select data samples from two databases for analysis, and use more advanced methods to reduce the errors caused by platforms, samples, etc., this is still a retrospective analysis. If we can carry out a prospective study or obtain clinical samples and evaluate them with Western blot or immunohistochemistry, it will be more convincing.

## Conclusion

We constructed an immune gene pair model which is closely related to the prognosis of cervical cancer patients. The model contains 29 IRGP and 47 immune-related genes. The biological functions of these 47 immune-related genes are closely related to the occurrence and development of cervical cancer. Therefore, we think that these IRGPs may be the target of predicting or diagnosing cervical cancer, and suggest that immunotherapy can improve the prognosis of cervical cancer patients by regulating these IRGPs.

## Data Availability

All data are available. Please contact us to access if it is needed.
